# HAP-Multitag, a PET and Positive MRI Contrast Nanotracer for the Longitudinal Characterization of Vascular Calcifications in Atherosclerosis

**DOI:** 10.1021/acsami.1c13417

**Published:** 2021-09-16

**Authors:** Juan Pellico, Irene Fernández-Barahona, Jesús Ruiz-Cabello, Lucía Gutiérrez, María Muñoz-Hernando, María J. Sánchez-Guisado, Irati Aiestaran-Zelaia, Lydia Martínez-Parra, Ignacio Rodríguez, Jacob Bentzon, Fernando Herranz

**Affiliations:** † CIBER de Enfermedades Respiratorias (CIBERES), 28029 Madrid, Spain; ‡ School of Biomedical Engineering & Imaging Sciences, King’s College London, St. Thomas’ Hospital, SE1 7EH London, U.K.; § Facultad de Farmacia, Universidad Complutense de Madrid, 28040 Madrid, Spain; ∥ Center for Cooperative Research in Biomaterials (CIC biomaGUNE), Basque Research and Technology Alliance (BRTA), 20014 Donostia San Sebastián, Spain; ⊥ IKERBASQUE, Basque Foundation for Science, 48013 Bilbao, Spain; # Departamento de Química Analítica, Instituto de Nanociencia y Materiales de Aragón, Universidad de Zaragoza-CSIC y CIBER-BBN, 50018 Zaragoza, Spain; ¶ Centro Nacional de Investigaciones Cardiovasculares Carlos III (CNIC), 28029 Madrid, Spain; ∇ NanoMedMol Group, Instituto de Química Medica (IQM), Consejo Superior de Investigaciones Científicas (CSIC), 28006 Madrid, Spain

**Keywords:** vascular calcifications, nanotracer, PET/MRI, hydroxyapatite, atherosclerosis

## Abstract

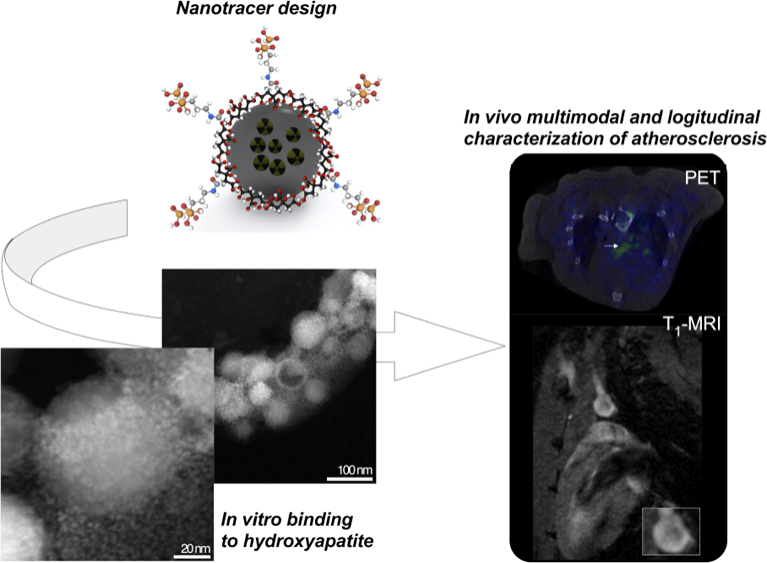

Vascular microcalcifications are associated with atherosclerosis plaque instability and, therefore, to increased mortality. Because of this key role, several imaging probes have been developed for their *in vivo* identification. Among them, [^18^F]FNa is the gold standard, showing a large uptake in the whole skeleton by positron emission tomography. Here, we push the field toward the combined anatomical and functional early characterization of atherosclerosis. For this, we have developed hydroxyapatite (HAP)-multitag, a bisphosphonate-functionalized ^68^Ga core-doped magnetic nanoparticle showing high affinity toward most common calcium salts present in microcalcifications, particularly HAP. We characterized this interaction *in vitro* and *in vivo*, showing a massive uptake in the atherosclerotic lesion identified by positron emission tomography (PET) and positive contrast magnetic resonance imaging (MRI). In addition, this accumulation was found to be dependent on the calcification progression, with a maximum uptake in the microcalcification stage. These results confirmed the ability of HAP-multitag to identify vascular calcifications by PET/(T_1_)MRI during the vulnerable stages of the plaque progression.

## Introduction

Atherosclerosis is a complex chronic inflammatory disease of the blood vessel wall in which plaques build up inside the arteries and is the leading cause of cardiovascular diseases. It is well known that the formation of calcified nodules is an important process in atherosclerosis development, often after a first inflammation step.^1^ These microcalcifications are associated with plaque rupture, leading to a cardiac event, or with plaque stabilization through the formation of macroscopic crystals (macrocalcifications) later in plaque development.^1,2^ Atherosclerosis microcalcifications are mainly composed of a mixture of hydroxyapatite (HAP), calcium oxalate monohydrate, and β-tricalcium phosphate, with HAP as the major component.^3^ Due to the relevance of these microcalcifications, several imaging probes have been developed in the past years. There are two main approaches to develop tracers for *in vivo* detection of calcifications: the use of [^18^F]FNa and bisphosphonate-based tracers. [^18^F]FNa is the gold standard for positron emission tomography (PET) detection of calcifications in the clinical scenario owing to the favorable pharmacokinetic profile and the lack of toxic effects.^4^ On the other hand, the [^18^F]FNa only binds to HAP, while bisphosphonate-based (BP) tracers or nanoparticles recognize a broader spectrum of calcium salts, relevant in several diseases.^5−8^ The mechanism of accumulation in calcifications is different for both types of tracers: in the case of [^18^F]FNa, ^18^F substitutes one hydroxyl group in the HAP matrix, forming fluorapatite, while when using BP-based probes, the bisphosphonate moiety coordinates with the Ca atom. When using [^18^F]FNa or BP-based tracers, one of the main drawbacks is the high uptake they show in the bone, increasing the off-target signal, often complicating vasculature differentiation.^9^ If the main focus is atherosclerosis, using a tracer for which the bone signal is minimized is highly desirable for imaging purposes. This limitation is overcome in humans and large animal models by selecting regions of interest (ROIs) in the imaging acquisition or post-processing steps. However, this strategy is impractical in small animal models where the vasculature is extremely small and PET resolution, even when combined with computed tomography (CT), is flawed. Examples of microcalcification detection in mice have been exclusively described in breast cancer and chronic tuberculous models.^10−12^

A second key aspect is the imaging modality. Current probes for vascular calcification detection are mainly based on nuclear imaging techniques, particularly PET. This technique offers unparalleled sensitivity but poor spatial resolution. For this reason, PET scanners are combined with CT and, more recently, with magnetic resonance imaging (MRI) scanners, providing detailed functional and anatomical information with micron resolution.^13^ The combination of PET with MRI is arguably the most convenient since it pieces together the extraordinary sensitivity of PET with the excellent resolution of MRI.^14,15^ The development of this technology is associated with the design of novel probes, providing signals in both imaging techniques. Among the different chemical compounds used to produce dual PET/MRI probes, iron oxide nanoparticles (IONPs) possess several advantages and one major drawback. IONPs are biocompatible and easy to produce, and there are a large variety of possible coatings to tune their bioconjugation and biodistribution.^16^ IONPs have a single drawback for this application; however, it is a major one: the typical signal they provide is T_2_-based, negative, or dark. This option complicates *in vivo* uptake identification, particularly in regions where an endogenous dark signal is present, like calcified vascular areas. This problem has drastically limited their use, especially in the clinical area, in molecular imaging or multimodal approaches. This void has boosted the quest for IONPs providing positive contrast in MRI, with several examples in the literature where positive contrast is achieved by tuning the core size,^17,18^ coating thickness,^19^ or core composition.^20^ Most of the time, the positive contrast is demonstrated by *in vivo* MR angiography. The dilution and sample redispersion in a large blood volume reduce the T_2_ effect, favoring the generation of positive contrast. However, examples of positive contrast in which IONPs accumulate in a specific tissue or organ are scarce.^20^

Here, we use bisphosphonate-based ^68^Ga-core-doped IONPs that we termed HAP-multitag, with several key features: they provide a simultaneous signal in PET and —positive contrast—MRI. HAP-multitag binds predominantly to HAP and other calcium salts relevant to vascular calcification, as demonstrated *in vitro* by different techniques. *In vivo*, HAP-multitag accumulation in atherosclerotic lesions can be monitored by PET and positive contrast MRI techniques. Finally, the accumulation is dependent on the stage of lesion development, which further demonstrates the ability of HAP-multitag to diagnose and longitudinally characterize atherosclerotic lesions by PET/(T_1_)MRI in mice.

## Results and Discussion

### Synthesis and Characterization of ^68^Ga-IONP-Alendronate

**Scheme 1:**
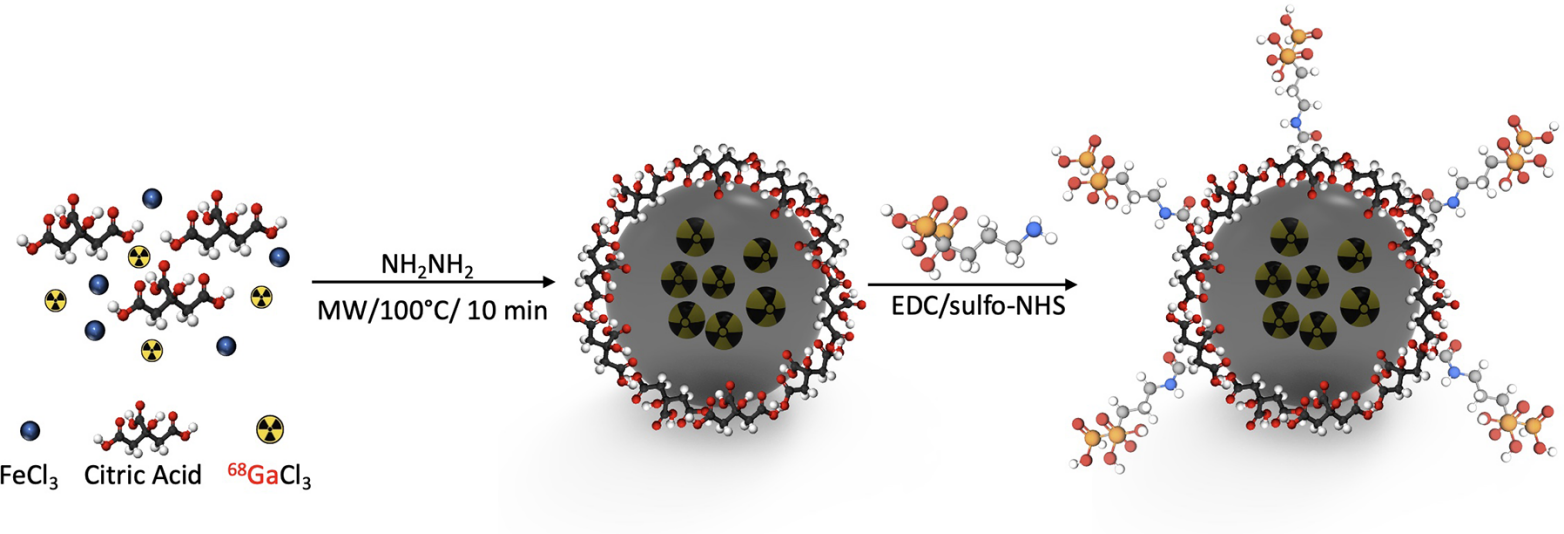
Microwave Two-Step Synthesis of ^68^Ga-IONP-Alendronate (HAP-Multitag Probe)

We synthesized HAP-multitag (^68^Ga-IONP-alendronate) in a two-step synthetic procedure (Scheme 1). First, a microwave-driven protocol rendered ^68^Ga-core-doped IONPs coated with citric acid (^68^Ga-IONP-citrate). This methodology, previously reported by our group, produces ^68^Ga-nanoparticles with high radiolabeling yield, high radiochemical purity and stability, and large *r*_1_ values, ensuring a remarkable response in both PET and positive contrast MRI.^21^ Then, we coupled the bisphosphonate moiety (alendronate sodium) by *N*-(3-dimethylaminopropyl)-*N*′-ethylcarbodiimide hydrochloride (EDC)/sulfo-NHS chemistry.^22^

**Figure 1:**
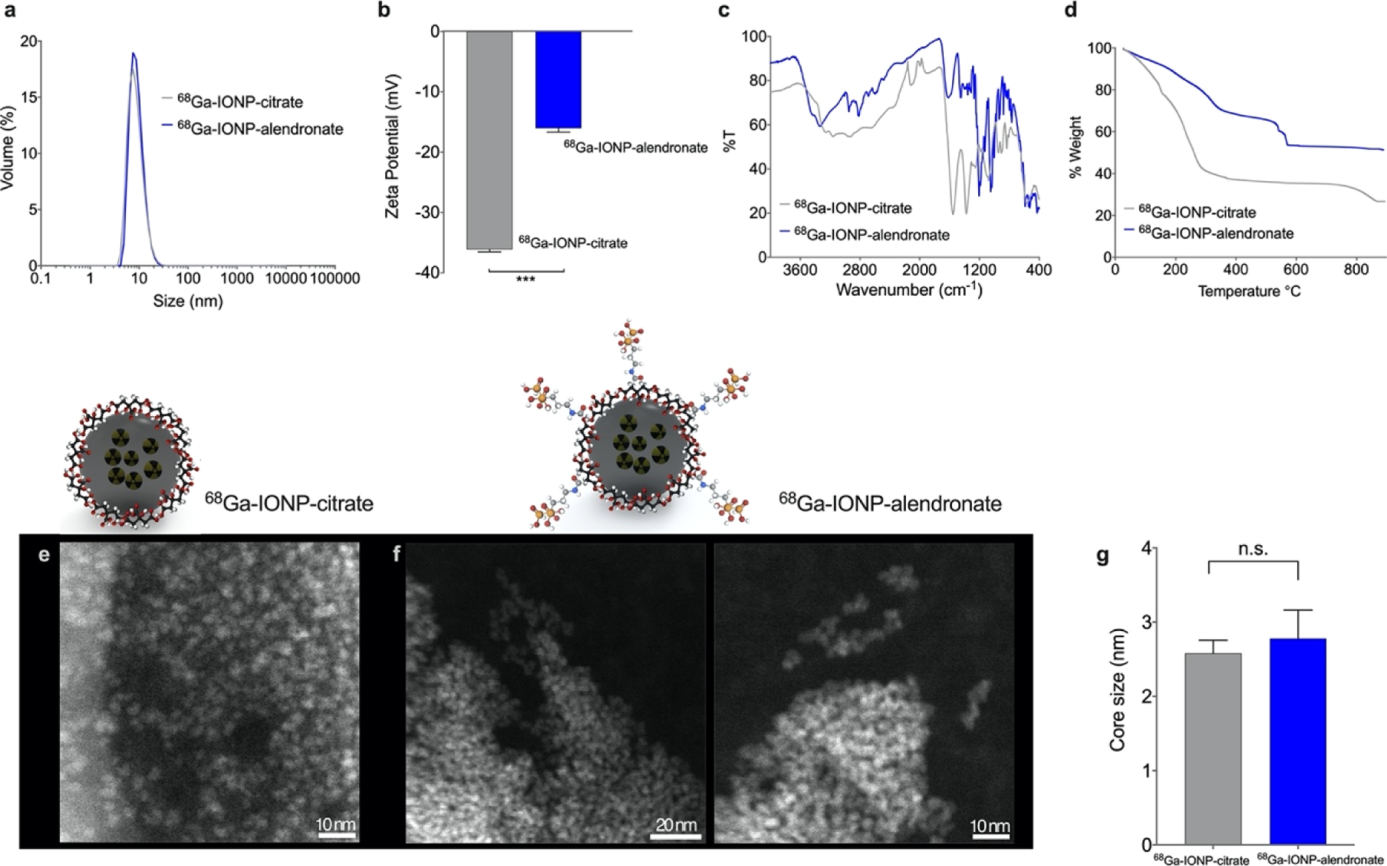
(a) DLS measurements for ^68^Ga-IONP-citrate and ^68^Ga-IONP-alendronate; (b) *Z*-potential (mV) of ^68^Ga-IONP-citrate and ^68^Ga-IONP-alendronate, *N* = 3, ***P < 0.001; (c) FTIR spectra of ^68^Ga-IONP-citrate and ^68^Ga-IONP-alendronate; (d) TGA of ^68^Ga-IONP-citrate and ^68^Ga-IONP-alendronate; (e) selected STEM-HAADF image of ^68^Ga-IONP-citrate; (f) selected STEM-HAADF image of ^68^Ga-IONP-alendronate; and (g) core size measured for ^68^Ga-IONP-citrate and ^68^Ga-IONP-alendronate. *N* = 50 per sample, n.s., *P* > 0.46.

After purification by size-exclusion chromatography, we analyzed the physicochemical properties of the radiolabeled nanoparticles. Dynamic light scattering (DLS) measurements show no differences between ^68^Ga-IONP-citrate and ^68^Ga-IONP-alendronate samples (Figure 1a), indicating no aggregation after the bioconjugation step, as expected for these hydrophilic nanoparticles when using EDC and sulfo-NHS as coupling agents. *Z*-potential measurement shows a significant reduction in the value of the superficial charge for ^68^Ga-IONP-alendronate (Figure 1b). The integration of the bisphosphonate moiety into the nanoparticle was confirmed by Fourier transform infrared (FTIR) spectroscopy (Figure 1c). The ^68^Ga-IONP-alendronate spectrum shows a new area with multiple peaks of strong intensity between 1250 and 900 cm^–1^ corresponding to the vibration modes of P=O and P–OH groups and new weaker peaks between 2700 and 2200 cm^–1^ attributed to the O–H stretches of the O=P–OH groups.^23^ Thermogravimetric analysis (TGA) (Figure 1d) further confirms the conjugation of alendronate to the surface of ^68^Ga-IONP-citrate, with the step between 540 and 590 °C corresponding to the covalent bond between citric acid and alendronate. According to TGA, the reduction in the organic coating, around 18%, can be attributed to the loss of citrate molecules from the surface in the second reaction and purification steps. This result (together with some exchange of citrate molecules by bisphosphonate moieties, exposing free amines) would also explain the reduction in the negative charge observed for ^68^Ga-IONP-alendronate in comparison to ^68^Ga-IONP-citrate. Using TGA information for ^68^Ga-IONP-alendronate, we calculated that each nanoparticle has been functionalized with approximately 140 molecules of alendronate. Finally, we studied ^68^Ga-IONP-citrate and ^68^Ga-IONP-alendronate by electron microscopy. Since these nanoparticles consist of an extremely small iron oxide core and a large organic coating, electron microscopy images are not easily obtained.

Using scanning transmission electron microscopy high-angle annular dark-field imaging (STEM-HAADF), it is possible to observe the small iron oxide cores without apparent aggregation for ^68^Ga-IONP-citrate (Figure 1e) and ^68^Ga-IONP-alendronate (Figure 1f). Analysis of the core sizes shows similar sizes for both nanoparticles, 2.6 ± 0.3 nm for ^68^Ga-IONP-citrate, and 2.8 ± 0.7 nm for ^68^Ga-IONP-alendronate.

### Qualitative Assessment of the Binding between ^68^Ga-IONP-Alendronate and Calcium Salts

**Figure 2:**
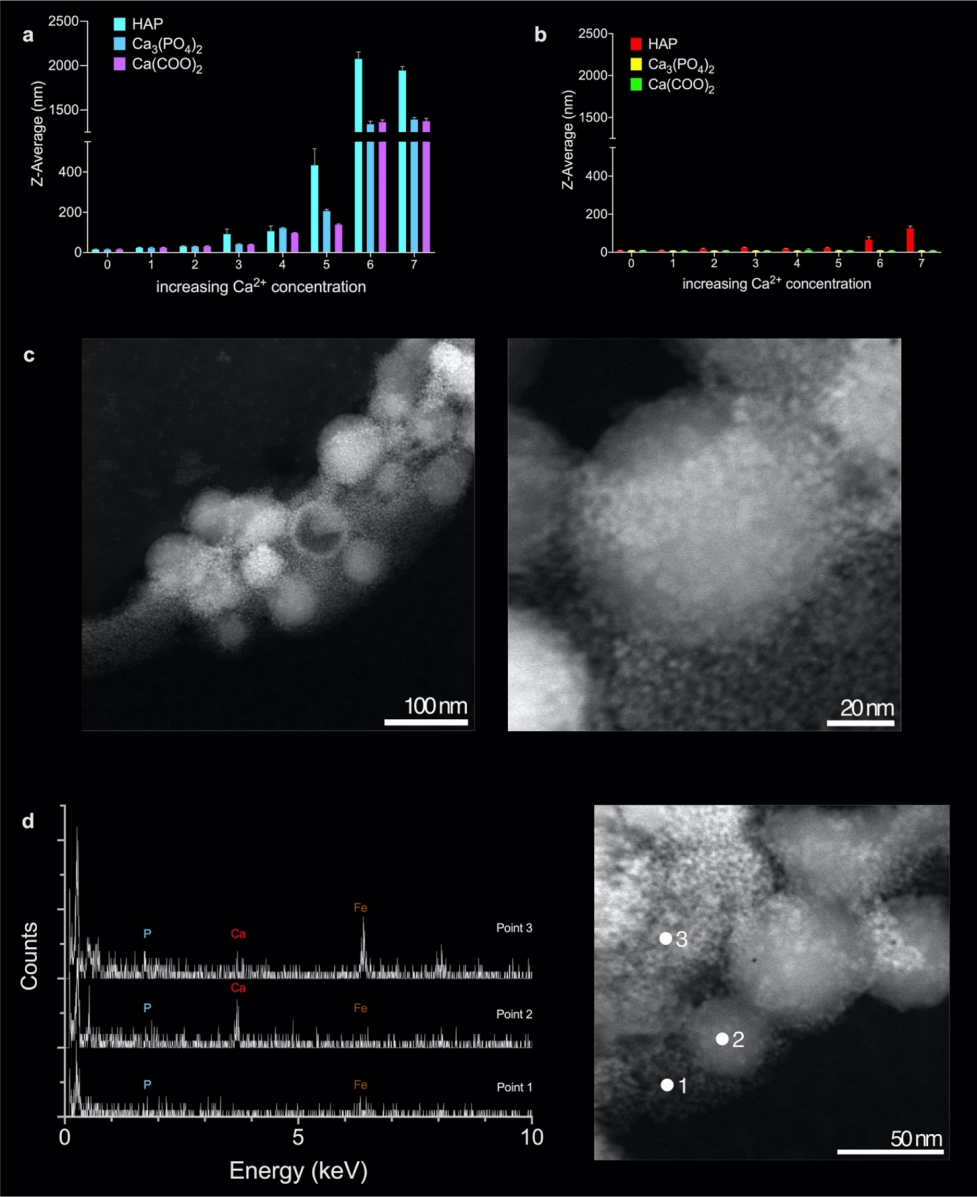
(a) Change in hydrodynamic size for ^68^Ga-IONP-alendronate upon the increase in the concentration of HAP, β-tricalcium phosphate, and calcium oxalate monohydrate; (b) change in hydrodynamic size for ^68^Ga-IONP-citrate upon the increase in the concentration of HAP, β-tricalcium phosphate, and calcium oxalate monohydrate; (c) STEM-HAADF images for the combination of ^68^Ga-IONP-alendronate with HAP; (d) EDX spectra for the three points indicated, in red, in the STEM-HAADF image.

To assess the interaction between ^68^Ga-IONP-alendronate and calcium salts, we chose those normally present in the microcalcifications structure, that is, HAP, calcium oxalate monohydrate, and β-tricalcium phosphate. First, we used DLS; by measuring the hydrodynamic size of the nanoparticles with increasing amounts of the calcium salts, it is possible to assess whether they are interacting or not.^24,25^ We incubated ^68^Ga-IONP-alendronate with the aforementioned salts and measured their hydrodynamic size (Figure 2a), and a similar procedure was followed with ^68^Ga-IONP-citrate (Figure 2b). The *Z*-average value clearly shows the aggregation of ^68^Ga-IONP-alendronate as the concentration of each salt increases. This is particularly true for HAP, which shows a very large hydrodynamic size value, around 2000 nm, for the highest concentration of the calcium salt. Similarly, the interaction with the other two salts, Ca_3_(PO_4_)_2_ and Ca(COO)_2_, is clearly reflected in the aggregation of the nanoparticles. As a control, we performed the same titrations but using ^68^Ga-IONP-citrate. In this case (Figure 2b), there is no aggregation when using the same calcium salts, as reflected in the constant value of hydrodynamic size (Figure 2a,b has the same scale in the *Y*-axis for better comparison). In fact, the size measured for the highest concentration of HAP with ^68^Ga-IONP-citrate reflects the presence of the HAP nanoparticles (with a size around 300 nm) rather than an interaction between the calcium salt and the citrate nanoparticles. This result is further demonstrated by the quantitative analysis (see below).

Then, we used STEM-HAADF to analyze the interaction between ^68^Ga-IONP-alendronate and HAP. Figure 2c shows the large HAP particles surrounded by the much smaller, ^68^Ga-IONP-alendronate nanoparticles, indicating their affinity toward the salt (more images in Figure S1). Zooming in the image, it is possible to see a single HAP particle completely surrounded by the much smaller ^68^Ga-IONP-alendronate nanoparticles. This was further confirmed by energy-dispersive X-ray microanalysis (EDX) analysis. Analyzing three different points, we can see the presence of Fe and P, when only ^68^Ga-IONP-alendronate is studied (point 1), the presence of large amounts of Ca when HAP with few surrounding ^68^Ga-IONP-alendronate particles is studied (point 2), also with Fe and P, and finally the presence of Fe, Ca, and P, in point 3, where many aggregated ^68^Ga-IONP-alendronate nanoparticles surround an HAP particle.

### Relaxometry

**Figure 3:**
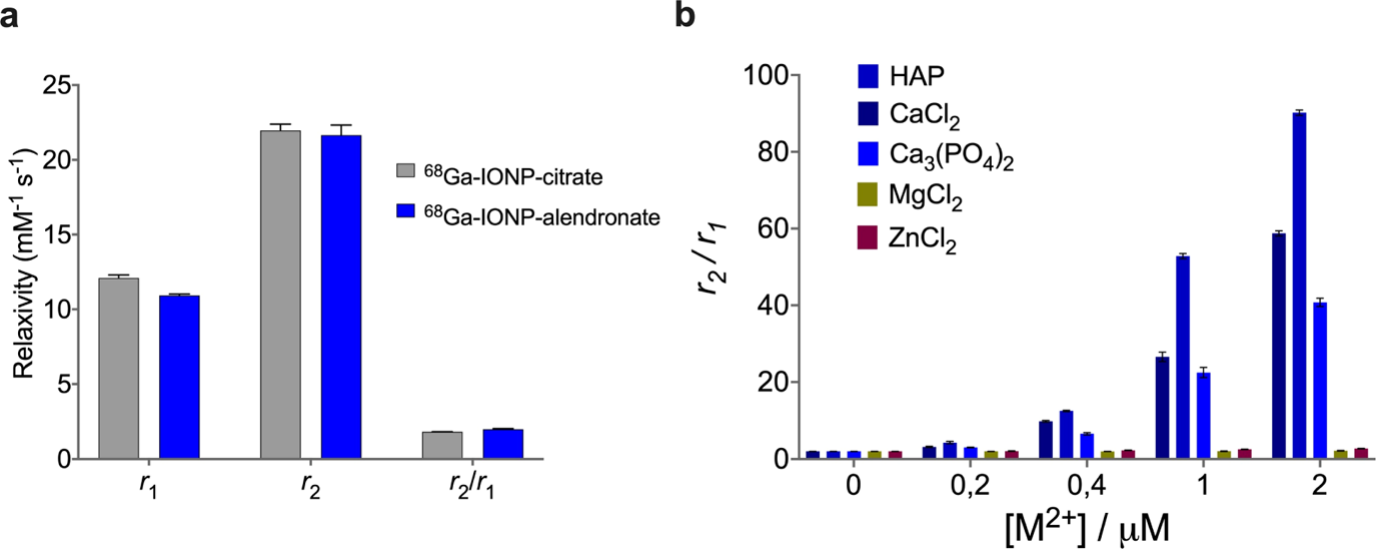
(a) *r*_1_, *r*_2_, and *r*_2_/*r*_1_ ratio for ^68^Ga-IONP-citrate and ^68^Ga-IONP-alendronate and (b) *r*_2_/*r*_1_ ratio of ^68^Ga-IONP-alendronate incubated with different concentrations of calcium, magnesium, and zinc salts.

Relaxometry of ^68^Ga-IONP-alendronate was carried out to confirm the positive contrast capabilities of the nanotracer and to assess the selectivity toward Ca^2+^ salts. Figure 3a shows the *r*_1_, *r*_2_, and *r*_2_/*r*_1_ values for ^68^Ga-IONP-citrate and ^68^Ga-IONP-alendronate, measured at 1.5 T. As expected, both nanotracers show positive contrast features with large *r*_1_ values and small *r*_2_ values, which produces *r*_2_/*r*_1_ values smaller than 2. At 1.5 T, the ^68^Ga-IONP-alendronate *r*_1_ value was 10.9 ± 0.1 mM^–1^ s^–1^, while the *r*_2_ value was 22.0 ± 0.4 mM^–1^ s^–1^, rendering a *r*_2_/*r*_1_ ratio of 1.98 ± 0.05. Figure 3b shows the *r*_2_/*r*_1_ ratio for ^68^Ga-IONP-alendronate as a function of the metal concentration for different salts. As expected, titration with Ca^2+^ salts produces an increase in the *r*_2_/*r*_1_ due to the aggregation of the nanotracer; while this reduces the T1 capabilities of the nanotracer, it would only be a problem for concentrations much larger than those we can find *in vivo*, as will be shown in the MRI experiments. Finally, the *r*_2_/*r*_1_ values when using Mg^2+^ or Zn^2+^ remain unchanged, confirming the well-known selectivity of bisphosphonate-functionalized nanoparticles toward Ca^2+^.

### Quantitative Assessment of the Binding between ^68^Ga-IONP-Alendronate and Calcium Salts

Next, we quantitively assessed the interaction between ^68^Ga-IONP-alendronate and ^68^Ga-IONP-citrate with the three calcium salts often present in vascular calcifications: HAP, Ca_3_(PO_4_)_2_, and Ca(COO)_2_ (Figure 3). For this, we covalently attached to the surface of the nanotracers a fluorescent dye (Alexa 647). The nanotracers were incubated with the different salts and purified by ultrafiltration, and the fluorescence of the supernatant was quantified at each point. The percentage of binding was calculated using the initial and final fluorescence intensities of the supernatant (see the Experimental Section).

**Figure 4:**
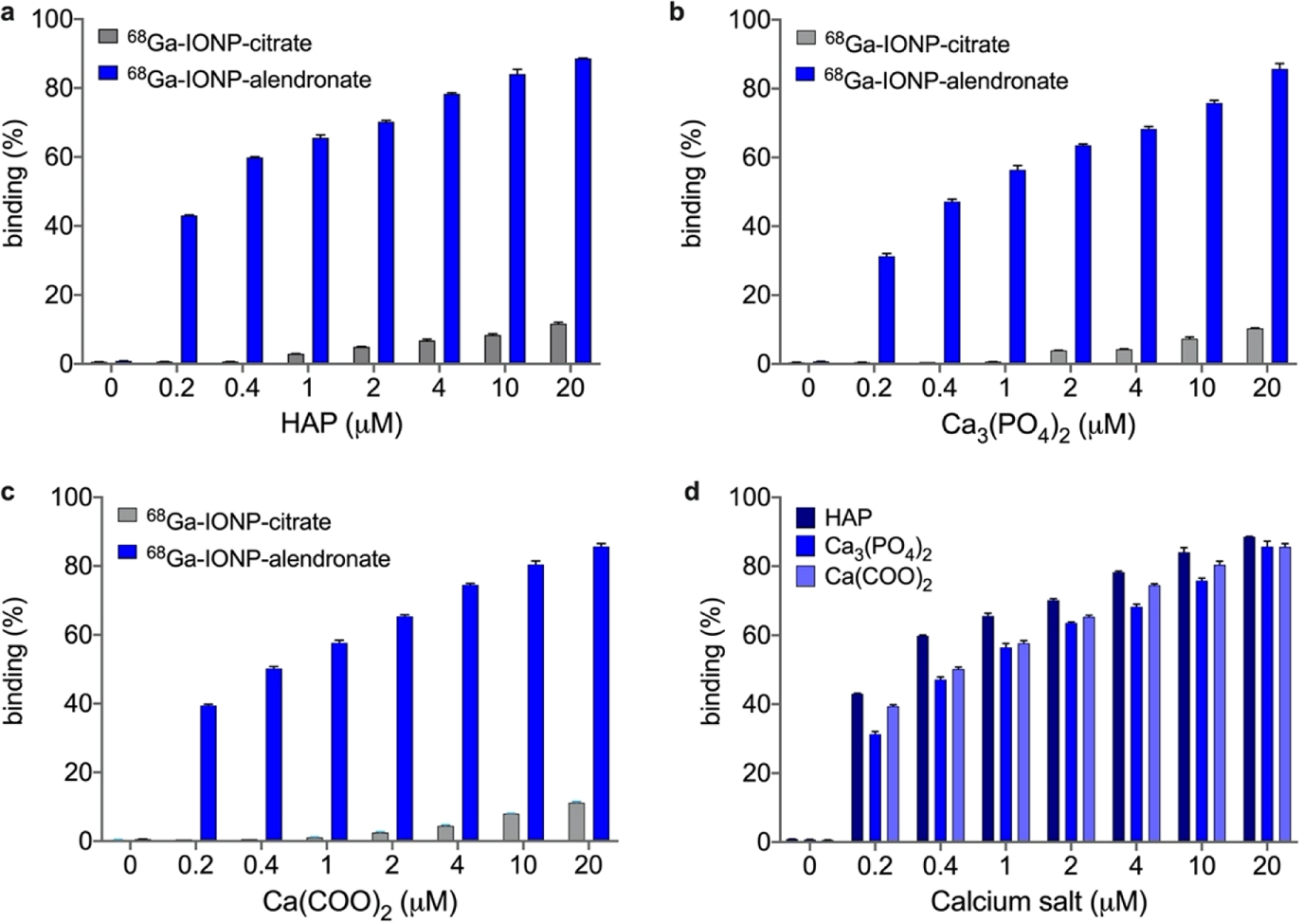
Percentage of binding between ^68^Ga-IONP-citrate and ^68^Ga-IONP-alendronate and (a) HAP, (b) Ca_3_(PO_4_)_2_, and (c) Ca(COO)_2_; (d) comparison of the binding between ^68^Ga-IONP-alendronate and the three calcium salts (60 min of incubation).

These titrations confirm several aspects: first, in agreement with the DLS data, the interaction between ^68^Ga-IONP-citrate and the different salts is negligible, a mere 11% for the largest HAP concentration (20 μM). On the contrary, titrations with ^68^Ga-IONP-alendronate clearly show a strong interaction, explaining the large aggregation observed in DLS and electron microscopy. For example, for a low concentration of HAP of 0.2 μM, the percentage of binding is already 43%; almost half of the nanotracer sample has bound the salt at this concentration (Figure 4a). Similarly, for the other salts (Figure 4b,c), there is large binding of the alendronate nanotracer without an appreciable interaction with the citrate nanoparticles. Finally, a similar profile was observed for the interaction of ^68^Ga-IONP-alendronate with Ca_3_(PO_4_)_2_ and ^68^Ga-IONP-alendronate with Ca(COO)_2_ (Figure 4d), with a slightly stronger interaction with HAP. For example, for the lowest calcium concentration, the percentages of binding are 43% with HAP, 31% with Ca_3_(PO_4_)_2_, and 39% with Ca(COO)_2_, confirming that this nanotracer presents a broad spectrum of interactions with calcium salts and not just limited to HAP, as is the case for [^18^F]FNa.

### Biodistribution of ^68^Ga-IONP-Alendronate, HAP-Multitag

**Figure 5:**
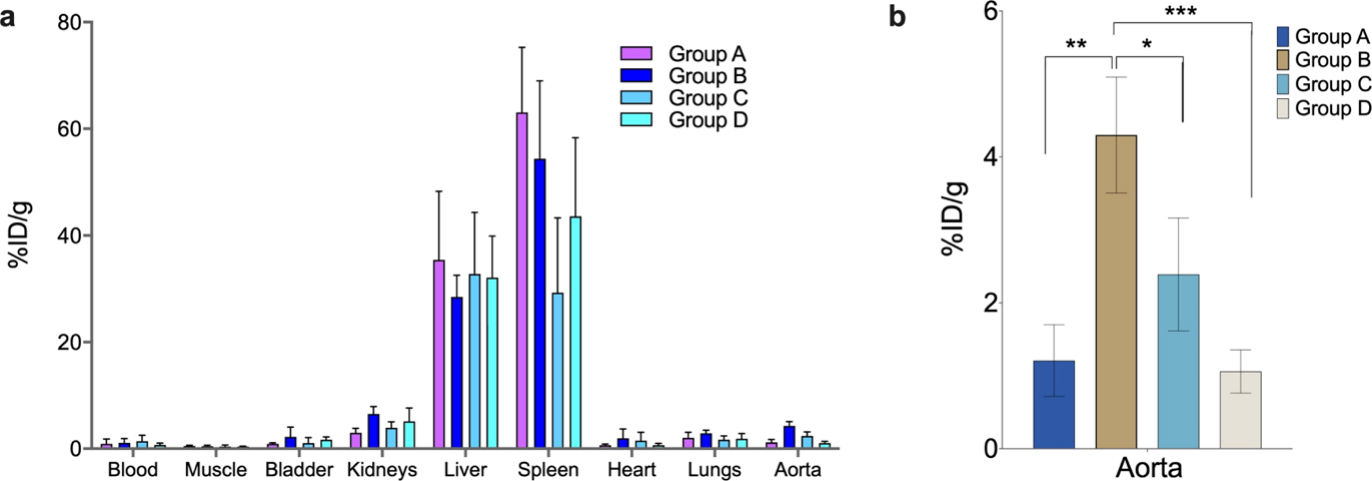
(a) Distribution of HAP-multitag measured in a gamma counter expressed as the percentage injected dose per gram (%ID/g) in ApoE^–/–^ mice (*N* = 5) of groups A–D; (b) aorta uptake of HAP-multitag showing significant differences between mice groups. *P < 0.05, **P < 0.01, ***P < 0.001, one-way ANOVA; error bars indicate s.d., *N* = 5.

After characterizing the *in vitro* interaction between ^68^Ga-IONP-alendronate and the selected calcium salts, we tested its performance to diagnose atherosclerosis. First, biodistribution experiments were conducted to evaluate whether HAP-multitag has any affinity toward atherosclerotic plaques in mice. Atherosclerotic ApoE^–/–^ mice were selected as the disease model. The development of hypercholesterolemia triggering aortic and carotid artery lesions throughout ApoE^–/–^ mice aging and high fat diet is well established.^26^ A longitudinal study was carried out in mice between 12 and 26 weeks of age. In addition, mice were fed with high-cholesterol diet from 8 weeks old onward to accelerate atherosclerosis progression.^27^ A complete biodistribution study was performed in a gamma counter after intravenous injection of ^68^Ga-IONP-alendronate in ApoE^–/–^ mice. We studied four different groups: 12 weeks old and fed 4 weeks with a high-fat cholesterol diet (HFD) (group A), 16 weeks old and 8 weeks HFD (group B), 24 weeks old and 16 weeks HFD (group C), and 26 weeks old and 18 weeks HFD (group D) (Figure 5a). Main organs, blood, and perfused aortas were evaluated in five mice of each group. Uptake values, calculated as the percentage of injected dose per gram of tissue, showed the liver and spleen as the organs with the highest accumulation. This is an expected result since biodistribution and clearance studies of IONPs have demonstrated the liver and spleen as the main uptake organs.^28^

Aortas show an important uptake with significant differences following mice aging and hence atherosclerosis progression (Figure 5b). Negligible blood circulation of the nanoparticles (<1.5 %ID/g) and the aortas’ perfusion, prior to the measurement, ensure that the signal measured in the aorta is due to nanoparticle uptake. Figure 5b shows the uptake of the nanoparticles in the aorta depending on the mice’s age. The HAP-multitag uptake is similar for the youngest and oldest mice, with a maximum for 16 weeks old mice. This observation may have important consequences for atherosclerosis characterization. First, this profile appears to follow the reported calcification process: initially, the amount of microcalcifications is too low to show a significant uptake—at 12 weeks; then, as more microcalcifications accumulate, an increase in the nanotracer uptake is observed—at 16 weeks; finally, the growth of the calcified deposits, and the concurrent reduction of the active surface, translates in a reduction of the nanotracer uptake, a process well known for other tracers.^4^ Similarly, [^18^F]FNa uptake appears to be inversely dependent on calcification growth.^29,30^ Second, the maximum uptake for HAP-multitag is the earliest reported, allowing for very early diagnosis of atherosclerosis. For comparison, the maximum uptake for [^18^F]FNa is reported in ApoE^–/–^ mice at 30 weeks old under high fat diet.^31^

### *In Vivo* Multimodal Imaging of Atherosclerosis with HAP-Multitag

#### PET/CT Imaging

**Figure 6:**
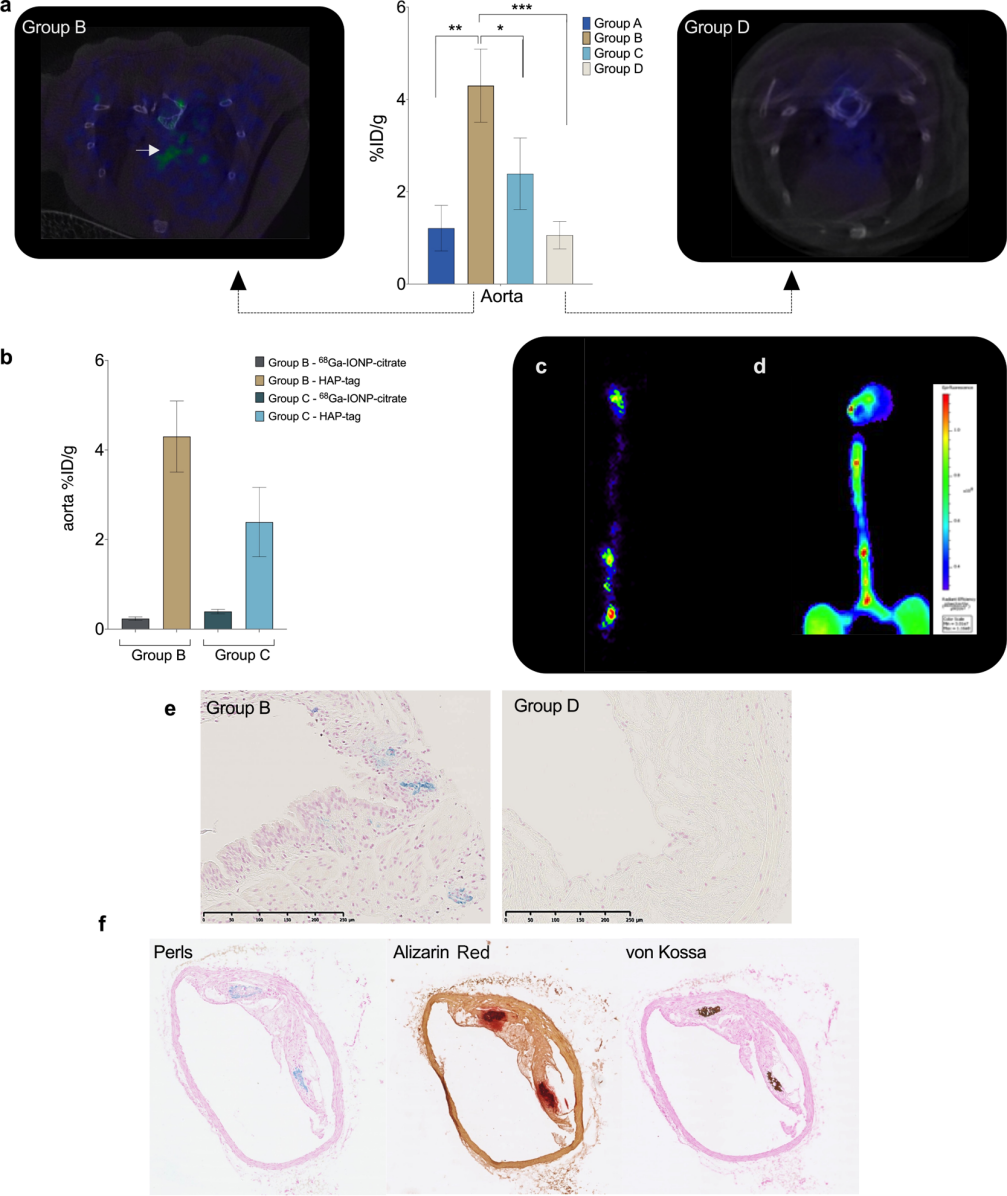
(a) Aorta uptake of HAP-multitag (the graph corresponds to Figure 5b, included here to compare uptake with images) and PET/CT images of group B and group D ApoE^–/–^ mice 90 min post i.v. injection with HAP-multitag; (b) uptake of nanotracers, expressed as the percentage injected dose per gram (%ID/g), in ApoE^–/–^ mice (*N* = 5) of groups B and C using ^68^Ga-IONP-citrate or HAP-multitag; error bars indicate s.d., *N* = 5. (c) *Ex vivo* PET imaging of a group B ApoE^–/–^ mouse aorta 90 min post i.v. injection with HAP-multitag; (d) *ex vivo* fluorescence imaging of a group B ApoE^–/–^ mouse aorta 24 h post i.v. injection of OsteoSense 680EX; (e) Perls’ Prussian Blue staining of aorta sections from group B and group D ApoE^–/–^ mice, both injected with HAP-multitag (scale bar is 250 μm). (f) Histology of the Group B ApoE^–/–^ mice aortas stained with Perls’ Prussian Blue, Alizarin Red, and von Kossa.

Encouraged by the biodistribution results, we tested the ability of the HAP-multitag probe to diagnose atherosclerosis by *in vivo* imaging by first using PET/CT. HAP-multitag was intravenously injected in group B ApoE^–/–^ mice, and images recorded 90 min post injection. Spots of nanotracer uptake are observed in the aortic arch and the aorta (Figures 6a and S2). PET/CT images were also obtained in group D ApoE^–/–^ mice for comparison. Contrary to what we see in young mice, these mice showed negligible uptake in the specific ROIs (Figure 6b), agreeing with the biodistribution results we have previously shown. Compared to [^18^F]FNa and other BP-based tracers, bone uptake of HAP-multitag is negligible (Figures 6a and S2). Uptake of HAP-multitag was also compared with the use of ^68^Ga-IONP-citrate as a nanotracer control. Figure 6b shows the percentage of injected dose per gram of tissue when using HAP-multitag or ^68^Ga-IONP-citrate in groups B and C ApoE^–/–^ mice (those groups with the highest uptake of the nanotracer). As expected, ^68^Ga-IONP-citrate does not accumulate in groups B and D ApoE^–/–^ mice since they lack the microcalcification targeting capabilities.

Nanotracer uptake was also confirmed by *ex vivo* PET imaging of excised aortas after *in vivo* experiments. Signaling spots are clearly identified throughout the aorta, predominantly in the aortic arch and the renal bifurcation (Figure 6c). To confirm whether the uptake is related to vascular calcifications, *ex vivo* fluorescence images were obtained using OsteoSense. This is a commercial dye, showing fluorescence in the near-infrared region, which includes a bisphosphonate moiety and is the gold standard for *ex vivo* microcalcification detection by fluorescence techniques.^32,33^ Following the manufacturer instructions, *ex vivo* fluorescence imaging was conducted 24 h post intravenous injection of OsteoSense in group B ApoE^–/–^ mice (*n* = 5, Figure S3). Comparing the *ex vivo* PET signal (Figure 6c) with the fluorescence signal from OsteoSense (Figure 6d), there is a perfect match between the different spots showing uptake of the probes, confirming the presence of microcalcifications in the sites where there is a clear uptake of HAP-multitag. The uptake of HAP-multitag in the aorta and its colocalization with microcalcification areas are further studied by histology. First, we compared the accumulation in aorta samples between groups B and D ApoE^–/–^ mice (Figures 6e and S4). While iron is clearly present in group B, as blue spots due to Perls’ Prussian Blue staining, there are no spots in group D. Then, we analyzed the colocalization between iron deposits in group B and microcalcifications to confirm the driving force for the uptake of the nanotracer. Figure 6f shows the results of triple stained aortas from a group B ApoE^–/–^ mouse. We detected iron accumulation with Perls and microcalcifications with Alizarin Red and von Kossa. The colocalization between iron deposits and microcalcifications is clearly visible (more images in Figure S5), confirming the mechanism of accumulation for HAP-multitag.

#### Magnetic Resonance Imaging

Finally, the HAP-multitag performance, as a positive contrast tracer in MRI, was evaluated. As extensively revised, nanoparticles with *r*_2_/*r*_1_ ratios below 4 have a high capability to provide positive MRI contrast.^17,19^ Therefore, the low ratio for HAP-multitag (Figure 3a) ensures its performance as a positive imaging probe in MRI. However, even with low ratios, the *in vivo* performance in large magnetic fields and with the accumulation in a particular tissue is more challenging.

**Figure 7:**
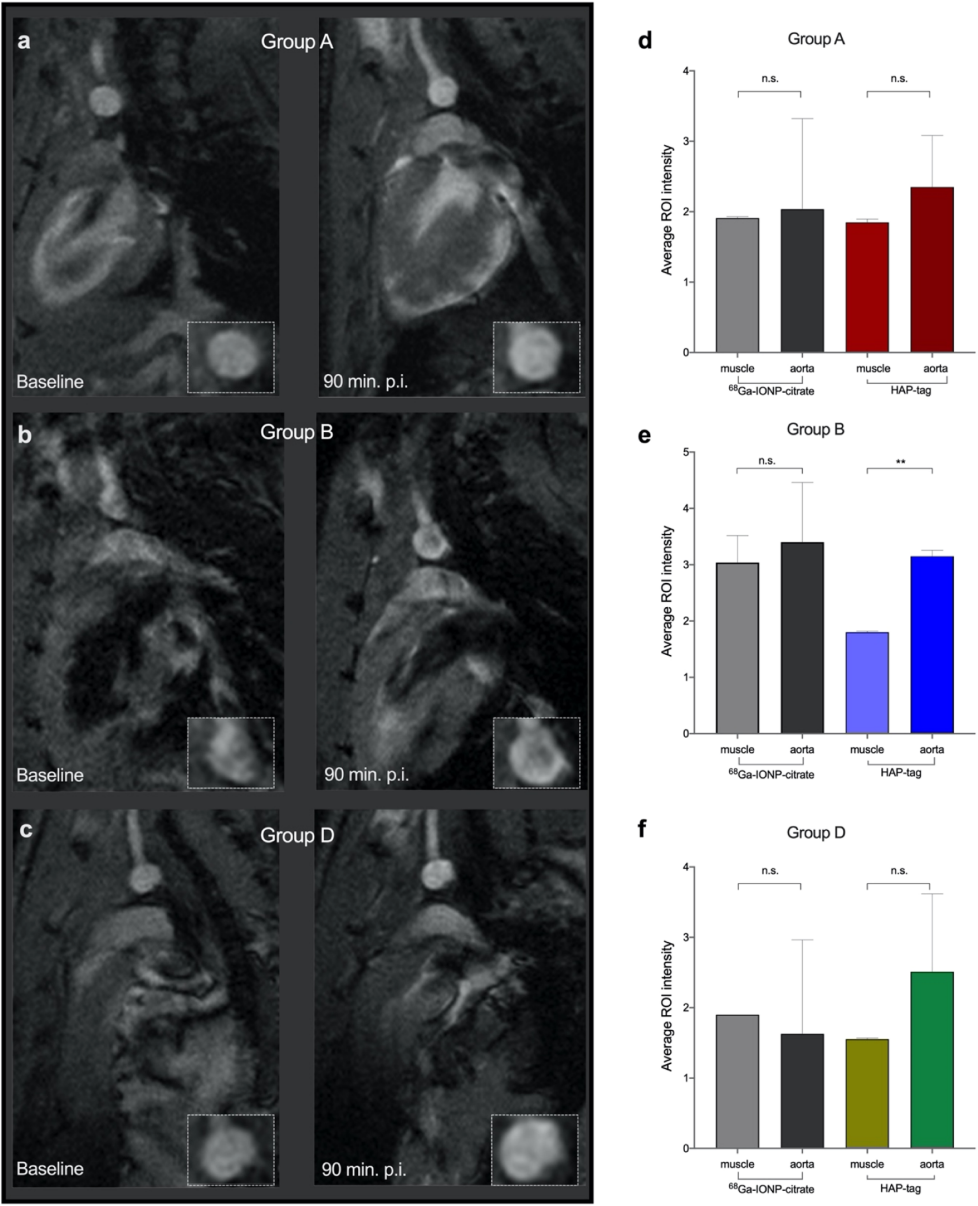
T_1_-weighted MRI before (baseline) and 90 min after i.v. injection of HAP-multitag for (a) group A ApoE^–/–^, (b) group B ApoE^–/–^, and (c) group D ApoE^–/–^; average ROI intensity (*n* = 10 per mouse) in the muscle and aorta 90 min after i.v. injection of ^68^Ga-IONP-citrate or HAP-multitag in ApoE^–/–^ mice (*n* = 3) for (d) group A ApoE^–/–^, (e) group B ApoE^–/–^, and (f) group D ApoE^–/–^. *P < 0.05, **P < 0.01, ***P < 0.001, one-way ANOVA; error bars indicate s.d.

*In vivo* imaging using non-functionalized ^68^Ga-IONP-citrate was conducted in group A, group B, and group D ApoE^–/–^ mice with no significant contrast enhancement observed in the aortic arch of these groups (Figures 7d–f and S6). Then, MRI was carried out using HAP-multitag as the nanotracer. In the case of group A and group D ApoE^–/–^ mice, some brightening of the arterial wall is visible; however, no significant contrast was observed (Figure 7a,c). On the contrary, the positive contrast was unambiguously appreciated 90 min after i.v. injection of HAP-multitag in the group B ApoE^–/–^ mice (Figures 7b and S7).

Semi-quantitative analysis of the images confirmed these results. For this, 10 different ROIs were selected in the muscle (used as reference) and the aorta in 3 different animals per group. No significant differences were obtained using ^68^Ga-IONP-citrate in all animals and HAP-multitag for group A and group D ApoE^–/–^ mice (Figure 7d–f). In agreement with the *in vivo* imaging results, significant intensity differences were found for group B ApoE^–/–^ mice. These results show the ability of HAP-multitag to generate positive contrast in MRI in a manner relevant to the calcification stage of the aorta.

## Conclusions

The development of PET/MRI as a powerful molecular imaging technique requires the development of imaging probes capable of providing simultaneous signals in both modalities. In this sense, IONPs are the perfect candidate due to their tailored synthesis, biofunctionalization, and biocompatibility. They are perfect for the purpose, with the exception of one key aspect, that is, the typical negative contrast they provide. Here, we show that it is possible to combine the PET signal and positive contrast using IONPs. The *in vitro* affinity of HAP-multitag for calcium salts translates into an *in vivo* uptake that depends on mice age and therefore in the calcification stage. We show how the targeted accumulation of these nanoparticles translates into easily identifiable PET and—bright signal—MRI beyond the magnetic resonance angiography typically performed with other IONPs. Using our nanotracer, HAP-multitag, it is possible to perform an early characterization of atherosclerotic plaques in ApoE^–/–^ mice just 16 weeks old. Its uptake enables the longitudinal characterization of microcalcifications.

## Experimental Section

^68^Ga (*t*_1/2_ = 68 min, β+ = 89%, and EC = 11%) was obtained from a ^68^Ge/^68^Ga generator system (ITG Isotope Technologies Garching GmbH, Germany) in which ^68^Ge (*t*_1/2_ = 270 d) was attached to a column based on an organic matrix generator. ^68^Ga was eluted with 4 mL of 0.05 M hydrochloric acid. Iron(III) chloride, hydrazine monohydrate, *N*-(3-dimethylaminopropyl)-*N*′-ethylcarbodiimide hydrochloride, *N*-hydroxysulfosuccinimide sodium salt, and alendronate sodium salt were purchased from Sigma-Aldrich. Citric acid trisodium salt dihydrate was purchased from Acros organics. OsteoSense 680TM EX was purchased from PerkinElmer, and disposable PD-10 desalting salt columns were purchased from GE Healthcare Life Sciences and Amicon Ultra centrifugal filters from Merck Millipore.

### Synthesis of ^68^Ga-IONP-Citrate

FeCl_3_ × 6 H_2_O (75 mg, 0.28 mmol), sodium citrate hydrate (80 mg, 0.27 mmol), and 1280 MBq of ^68^GaCl_3_ in HCl (0.05 M, 4 mL) were dissolved in water (5 mL) in a microwave-adapted flask, followed by addition of 1 mL of hydrazine hydrate. The solution was ramped to 120 °C over 54 s and held at this temperature for 10 min (240 W) in a Monowave 300 microwave reactor equipped with an internal temperature probe and an external IR probe (Anton Paar, GmbH, Ostfildern-Scharnhausen, Germany). The reaction mixture was then cooled to 60 °C, and the ^68^Ga-IONP-citrate product was purified by passing the mixture through a PD-10 column to eliminate excess small reagents, including all unincorporated radioisotopes. This purification process provided 9 mL of ^68^Ga-IONP-citrate with a total activity of 781 MBq (measured 40 min after starting the reaction), with a radiolabeling yield of 92%.

### Synthesis of ^68^Ga-IONP-Alendronate (HAP-Multitag)

To 750 MBq of ^68^Ga-IONP-citrate (5 mL) were added 0.07 mmol of EDC and 0.075 mmol of *N*-hydroxysulfosuccinimide sodium salt (sulfo-NHS). The solution was stirred for 30 min at room temperature (r.t.) and then ultracentrifuged at 10,350*g* through Amicon Ultra-15 30 kDa centrifugal filters for 4 min to remove excess reagents. The retentate was resuspended in 1.5 mL of N-(2-hydroxyethyl)piperazine-N′-ethanesulfonic acid (HEPES) buffer, pH 8, and 1 mg of alendronate sodium salt was added to the solution. The mixture was maintained at r.t for 60 min with stirring. Finally, another ultrafiltration step was performed to eliminate unreacted alendronate. The retentate was resuspended in saline solution, giving 195.6 MBq of HAP-multitag with a radiolabeling yield of 98%.

### Physicochemical Characterization

The hydrodynamic size and polydispersity index were measured with a Zetasizer Nano ZS90 system (Malvern Instruments, UK) using folded capillary cells with samples in water unless other solvents are indicated. For determination of the morphology and mean particle size and distribution, samples were examined under a transmission electron microscope (Tecnai F30, FEI) operated at 300 kV using scanning-transmission imaging with a high-angle annular dark-field detector (STEM-HAADF). Chemical analysis of the nanoparticles was performed by EDX. A drop of the nanoparticle suspension was deposited onto a holey-carbon-coated copper grid and left to evaporate at r.t. Mean sizes and standard deviations were calculated for approximately 50 particles.

### Sample Preparation for Electron Microscopy

^68^Ga-IONP-citrate and ^68^Ga-IONP-alendronate (cold samples, without the active ^68^Ga isotope) were incubated with 20 μM HAP. After 30 min of incubation at r.t., a drop of the nanoparticle suspension was deposited onto a holey-carbon-coated copper grid and left to evaporate at r.t.

### Titration of the Ca^2+^ Salts

^68^Ga-IONP-citrate and ^68^Ga-IONP-alendronate (cold samples, without the active ^68^Ga isotope) were incubated with different concentrations (0.2, 0.4, 1, 2, 4, 10, 20 μM) of three different calcium salts: HAP, calcium oxalate monohydrate, and β-tricalcium phosphate. After 60 min of incubation at r.t., the hydrodynamic size of the samples was measured using a Zetasizer Nano ZS90 system (Malvern Instruments, UK).

### Binding Quantification by Fluorescence

The Alexa Fluor 647 (A647) dye (excitation λ = 649 nm; emission λ = 666 nm) was used to quantify the binding (%) of ^68^Ga-IONP-citrate and ^68^Ga-IONP-alendronate (cold samples, without the active ^68^Ga isotope) to different calcium salts: HAP, calcium oxalate monohydrate, and β-tricalcium phosphate. To synthesize ^68^Ga-IONP-citrate-A647 and ^68^Ga-IONP-alendronate-A647, 5 mL of ^68^Ga-IONP-citrate was added to 0.07 mmol EDC and 0.075 mmol of *N*-hydroxysulfosuccinimide sodium salt (sulfo-NHS). The solution was stirred for 30 min at r.t. and then ultracentrifuged at 10,350*g* through Amicon Ultra-15 30 kDa centrifugal filters for 4 min to remove excess reagents. The retentate was resuspended in 1.5 mL of HEPES buffer, pH 8, and 100 μg of Alexa 647 hydrazide to synthesize ^68^Ga-IONP-citrate-A647, and 100 μg of Alexa 647 hydrazide plus 1 mg of alendronate sodium salt to obtain ^68^Ga-IONP-alendronate-A647. The samples were maintained at r.t. for 60 min under vigorous stirring. Once this step was finished, samples were purified by ultrafiltration to eliminate unreacted A647 and alendronate. The retentate was resuspended in saline solution.

^68^Ga-IONP-citrate-A647 and ^68^Ga-IONP-alendronate-A647 were incubated for 60 min at r.t. with different concentrations of the calcium salts (0.2, 0.4, 1, 2, 4, 10, 20 μM). Posteriorly, supernatant fluorescence was measured at λ = 666 nm after 150 min centrifugation at 13,680*g*.

The degree of Ca salt binding was assessed using the following formula:
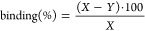
1where *X* is the initial amount of fluorescence in ^68^Ga-IONP-citrate-A647 and ^68^Ga-IONP-alendronate-A647 and *Y* is the amount of fluorescence left in the supernatant after centrifugation.

### Relaxometry

Relaxometric properties of the samples were assessed by measuring longitudinal and transverse relaxation times. Four concentrations of each nanoparticle sample were selected, and the longitudinal and transversal relaxation times of each one were measured using a Bruker mq60 at 1.5 T and 37 °C. The *r*_1_ and *r*_2_ values were plotted against the Fe concentration (0, 0.25, 0.5, 1, and 2 mM).

^68^Ga-IONP-citrate and ^68^Ga-IONP-alendronate (cold samples, without the active ^68^Ga isotope) were incubated with different concentrations (0.2, 0.4, 1, 2, 4, 10, 20 μM) of three different calcium salts: HAP, calcium oxalate monohydrate, and β-tricalcium phosphate, as well as magnesium and zinc chloride. After 60 min of incubation at r.t., *r*_1_ and *r*_2_ values were measured to calculate the *r*_2_/*r*_1_ ratio.

### Animal Model

Mice were housed in the specific pathogen-free facilities at the Centro Nacional de Investigaciones Cardiovasculares Carlos III, Madrid. All animal experiments conformed to EU Directive 2010/63EU and Recommendation 2007/526/EC, enforced in Spanish law under Real Decreto 53/2013. The protocol was approved by the Madrid regional government (PROEX16/277).

ApoE^–/–^ mice were fed with high-cholesterol diet (Western diet) from 8 weeks old onward to obtain the atherosclerosis mouse model.

### PET/CT Imaging

*In vivo* PET/CT imaging in mice was performed with a nanoPET/CT small-animal imaging system (Mediso Medical Imaging Systems, Budapest, Hungary). List-mode PET data acquisition commenced 90 min after injection of a bolus of 10–15 MBq of HAP-multitag through the tail vein and continued for 30 min. At the end of PET, a micro-CT was performed for attenuation correction and anatomic reference. The dynamic PET images were reconstructed in a 105 × 105 matrix (frame rates: 3 × 10 min, 1 × 30 min, 1 × 60 min) using a Tera-Tomo 3D iterative algorithm. Images were obtained and reconstructed with proprietary Nucline software (Mediso, Budapest, Hungary). Images were analyzed using Horos software v.3.3.6.

### Fluorescence Imaging

Experiments were conducted following the standard protocol provided by the manufacturer. OsteoSense 680EX was reconstituted by addition of 1.2 mL of phosphate-buffered saline (PBS) 1× into the vial. The mixture was gently shaken for 5 min at r.t. Then, 100 μL of the resultant solution was intravenously injected into 5 ApoE^–/–^ mice. 24 h post injection, animals were sacrificed in a CO_2_ chamber and perfused with 8 mL of PBS 1×, and the aortas were excised. *Ex vivo* imaging of the aortas was carried out in an IVIS Imaging System 200, Xenogen (acquisition parameters: Cy5.5 ex/em filter, high level, BIN-HR, FOV 13.3, f2, 4s).

### MRI Acquisition

All experiments were performed on a 7 T Bruker Biospec 70/30 USR MRI system (Bruker Biospin GmbH, Ettlingen, Germany), interfaced to an AVANCE III console. Anesthesia was induced with 3% isoflurane in 30% oxygen and maintained 1–2% isoflurane along the experiment.

A BGA12 imaging gradient (maximum gradient strength 400 mT/m) system with a 40 mm diameter quadrature volume resonator was used for MRI data acquisition. Animals were positioned in a customized 3D printed bed with a head holder and kept warmed with heated air pumped through an MRI compatible system interfaced to a Monitoring and Gating Model 1025 (SA instruments). Temperature control (anal) and respiration (through a respiratory pad) were registered along the experiment.

To ensure an accurate positioning, pure axial and four-chamber view scout images were used to set up the representative aortic arch view. From these, images were obtained between the brachiocephalic artery and left common carotid artery, perpendicular to the direction of the flow in the aorta. A single 0.8 mm, 2.8 × 2.8 cm isotropic FOV (obtained and reconstructed with 256 × 256) slice was obtained using a Bruker self-gated cine gradient echo FLASH sequence using the following parameters: minimum TE 4 ms, TR 9 ms, flip angle 10°, 1 average. An additional image in the same position was obtained with a fat suppression module.

### *Ex Vivo* Biodistribution

Biodistribution was studied with a Wizard 1470 gamma counter (PerkinElmer). Animals were sacrificed in a CO_2_ chamber, after which blood was extracted and the animals perfused with 8 mL of PBS 1×. Organs were extracted and counted in the gamma counter for 1 min each. Readings were decay-corrected and presented as the percentage of injected dose per gram (%ID/g).

### Histological Analysis

Excised aortas were fixed in 10% formalin for 24 h. The tissue was dehydrated and embedded in paraffin until sectioning. Aorta sections were stained with Perl’s Prussian Blue, von Kossa, and Alizarin red. Images were processed and digitalized with NIS-Elements acquisition software.

## Supporting Information Available

Additional STEM-HAADF, PET/CT, fluorescence, and MRI images (PDF)

## Abbreviations

PET: positron emission tomography
MRI: magnetic resonance imaging
HAP: hydroxyapatite
BP: bisphosphonate
CY: computed tomography
IONPs: iron oxide nanoparticles
EDC: *N*-(3-dimethylaminopropyl)-*N*′-ethylcarbodiimide hydrochloride
NHS: *N*-hydroxysuccinimide
DLS: dynamic light scattering
FTIR: Fourier transform infrared
STEM-HAADF: scanning transmission electron microscopy high-angle annular dark-field imaging
