# Supplementary material for: HAP-Multitag, a PET and Positive MRI Contrast Nanotracer for the Longitudinal Characterization of Vascular Calcifications in Atherosclerosis
Source: ACS Appl Mater Interfaces. 2021 Sep 16;13(38):45279–90. doi: 10.1021/acsami.1c13417 (PMC8485330; doi:10.1021/acsami.1c13417)
Supplement: Supplementary file 1 — am1c13417_si_001.pdf [file am1c13417_si_001.pdf]

Supporting information

HAP-multitag, a PET and positive MRI contrast nanotracer for the longitudinal characterization of vascular calcifications in atherosclerosis

Juan Pellico,[a,b‡] *Irene Fernández-Barahona,*[c,h‡] *Jesús Ruiz-Cabello,*[a,c,d,e] *Lucía Gutiérrez,*[f f] *María Muñoz-Hernando,*[h,g] *María J. Sánchez-Guisado,*[d] *Irati Aiestaran-Zelaia,*[d] *Lydia Martínez-Parra,*[d] *Ignacio Rodríguez,*[a,c] *Jacob Bentzon,*[g] *Fernando Herranz*[a,h] *

[a] CIBER de Enfermedades Respiratorias (CIBERES), 28029 Madrid, Spain.

[b] School of Biomedical Engineering & Imaging Sciences, King's College London, St. Thomas' Hospital, London SE1 7EH, UK

[c] Facultad de Farmacia, Universidad Complutense de Madrid, 28040 Madrid, Spain

[d] Center for Cooperative Research in Biomaterials (CIC biomaGUNE), Basque Research and Technology Alliance (BRTA), 20014 Donostia San Sebastián, Spain

[e] IKERBASQUE, Basque Foundation for Science, 48013 Bilbao, Spain

[f] Departamento de Química Analítica, Instituto de Nanociencia y Materiales de Aragón, Universidad de Zaragoza-CSIC y CIBER-BBN, 50018 Zaragoza, Spain

[g] Centro Nacional de Investigaciones Cardiovasculares Carlos III (CNIC), 28029 Madrid, Spain.

[h] NanoMedMol Group, Instituto de Química Medica (IQM), Consejo Superior de

Investigaciones

Científicas (CSIC), 28006 Madrid, Spain

E-mail address: fherranz@iqm.csic.es

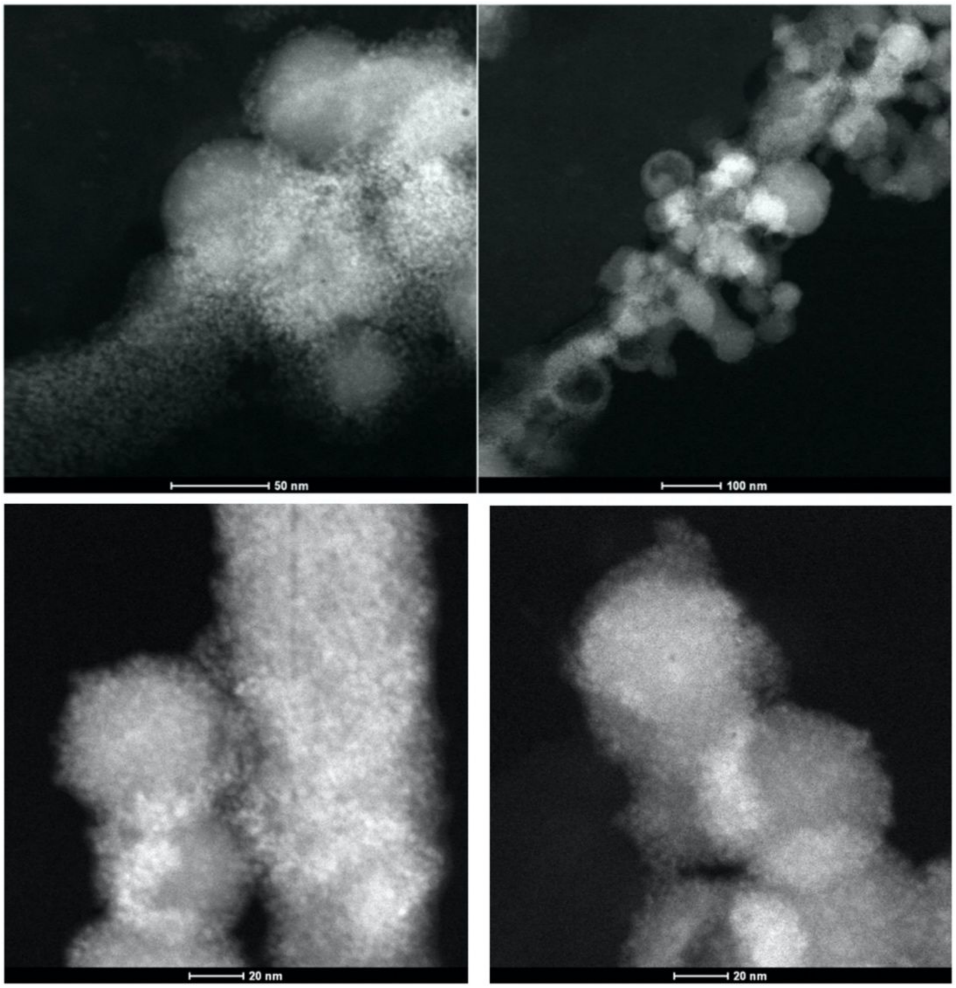

Figure S1. STEM-HAADF images for the combination of ^{68}Ga-IONP-alendronate.

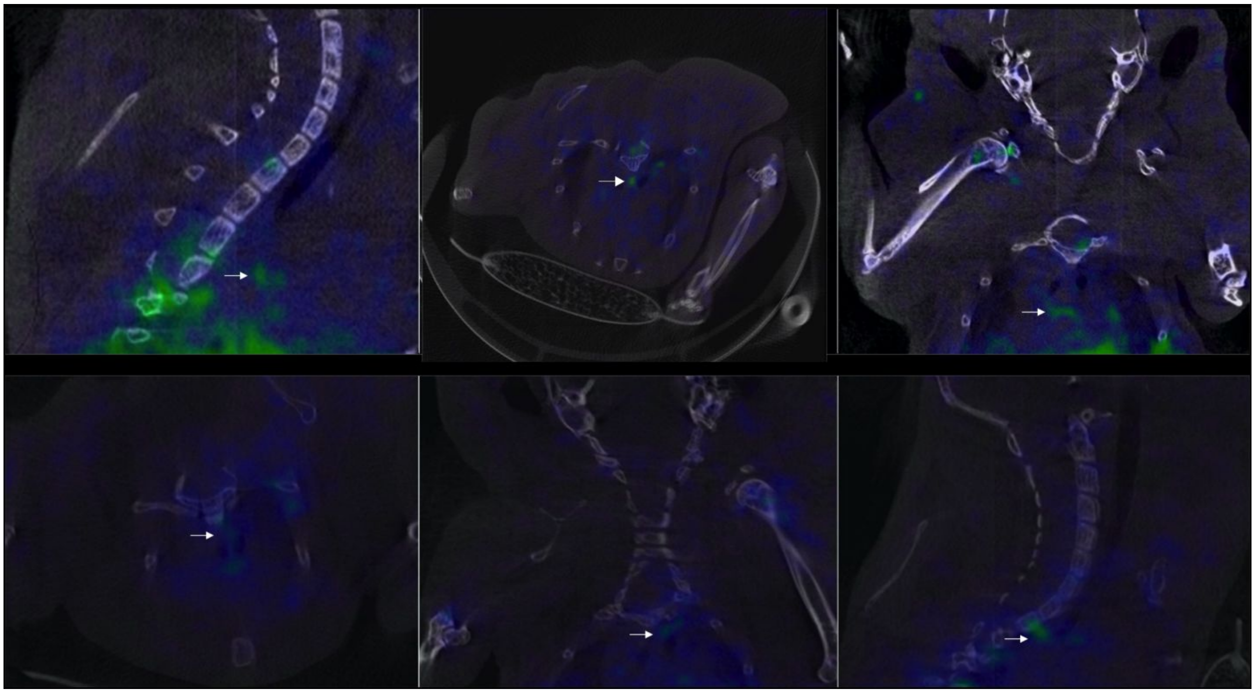

Figure S2. PET/CT imaging of a Group B ApoE$^{-/-}$ mice 90 min post *i.v.* injection with HAP-multitag

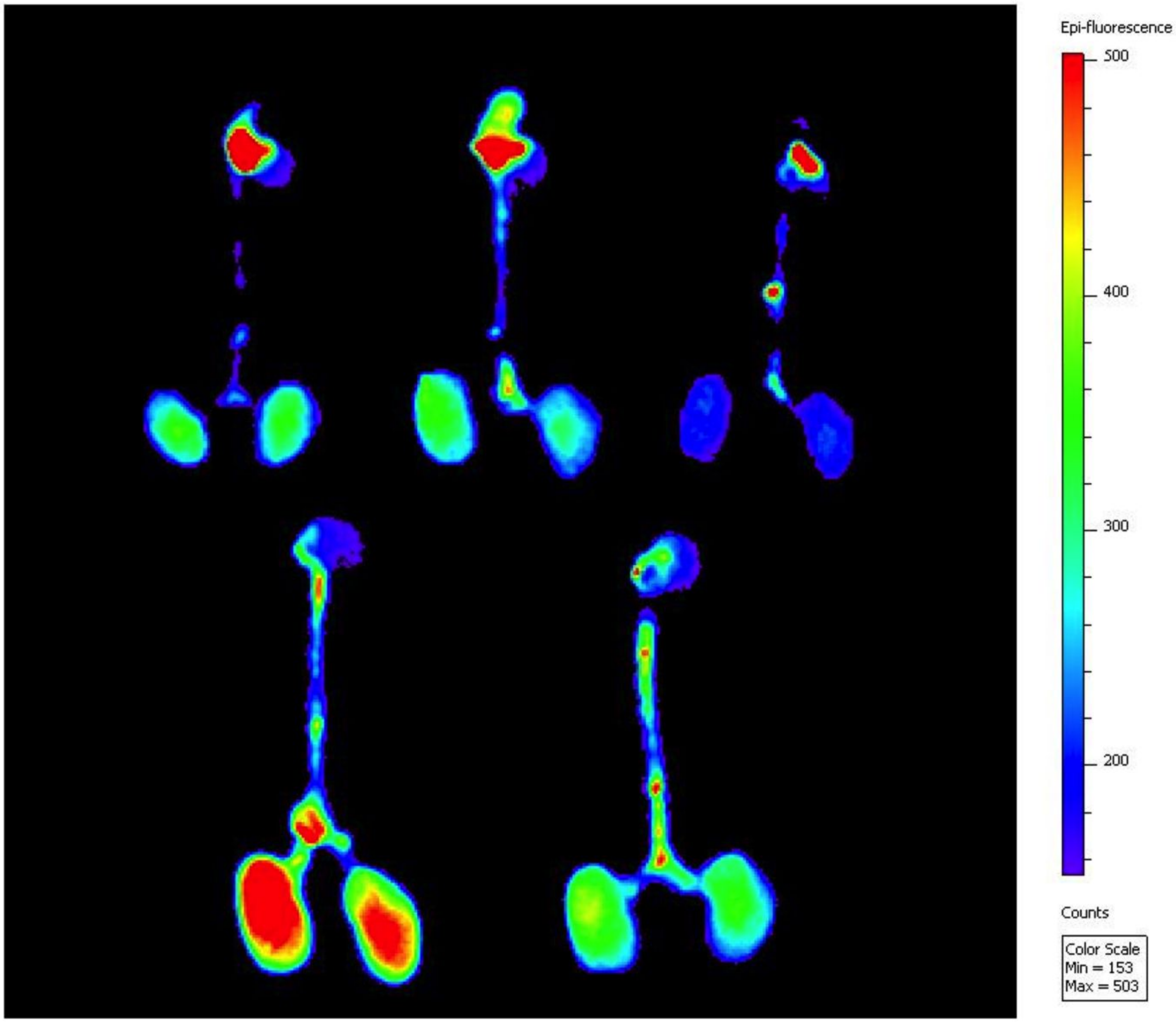

Figure S3. *Ex vivo* fluorescence imaging of 5 mice aortas 24 h post *i.v.* injection of OsteoSense® 680EX in Group B ApoE$^{-/-}$ mice.

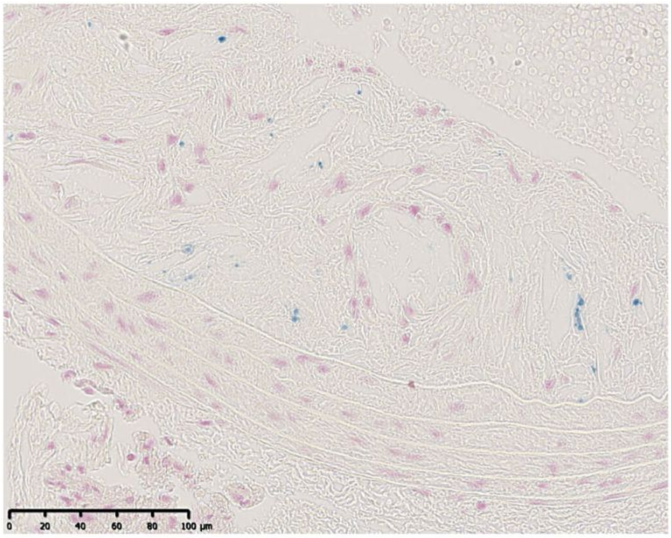
0 20 40 60 80 100 µm

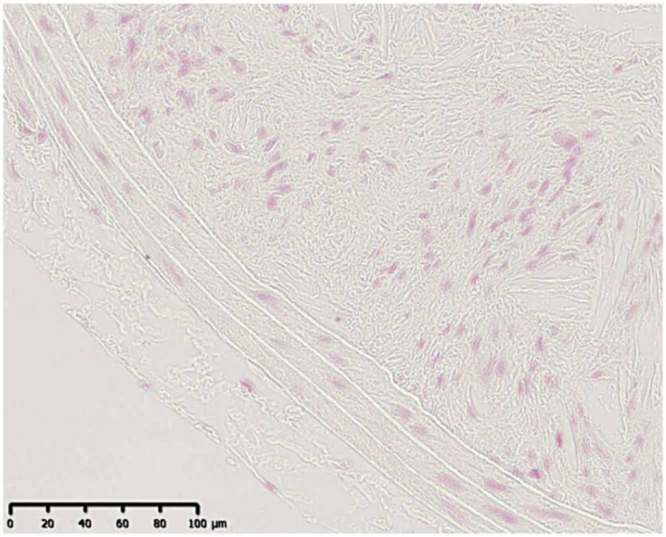
0 20 40 60 80 100 µm

Figure S4. Perls' Prussian blue staining of aorta sections from a Group B $ApoE^{-/-}$ mouse (top row) and a Group D $ApoE^{-/-}$ mouse (bottom row), both injected with HAP-multitag (scale bar is 100 μm).

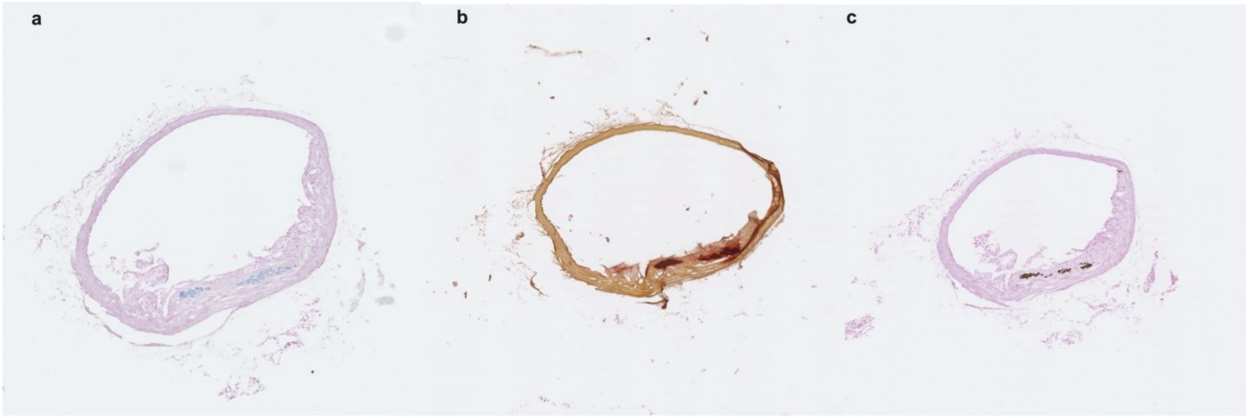

Figure S5. Histology of the Group B $ApoE^{-/-}$ mice aortas stained with Perls' Prussian Blue (a), alizarin red (b) and von Kossa (c). Colocalization between the presence of iron (a) and microcalcifications (b and c) is shown.

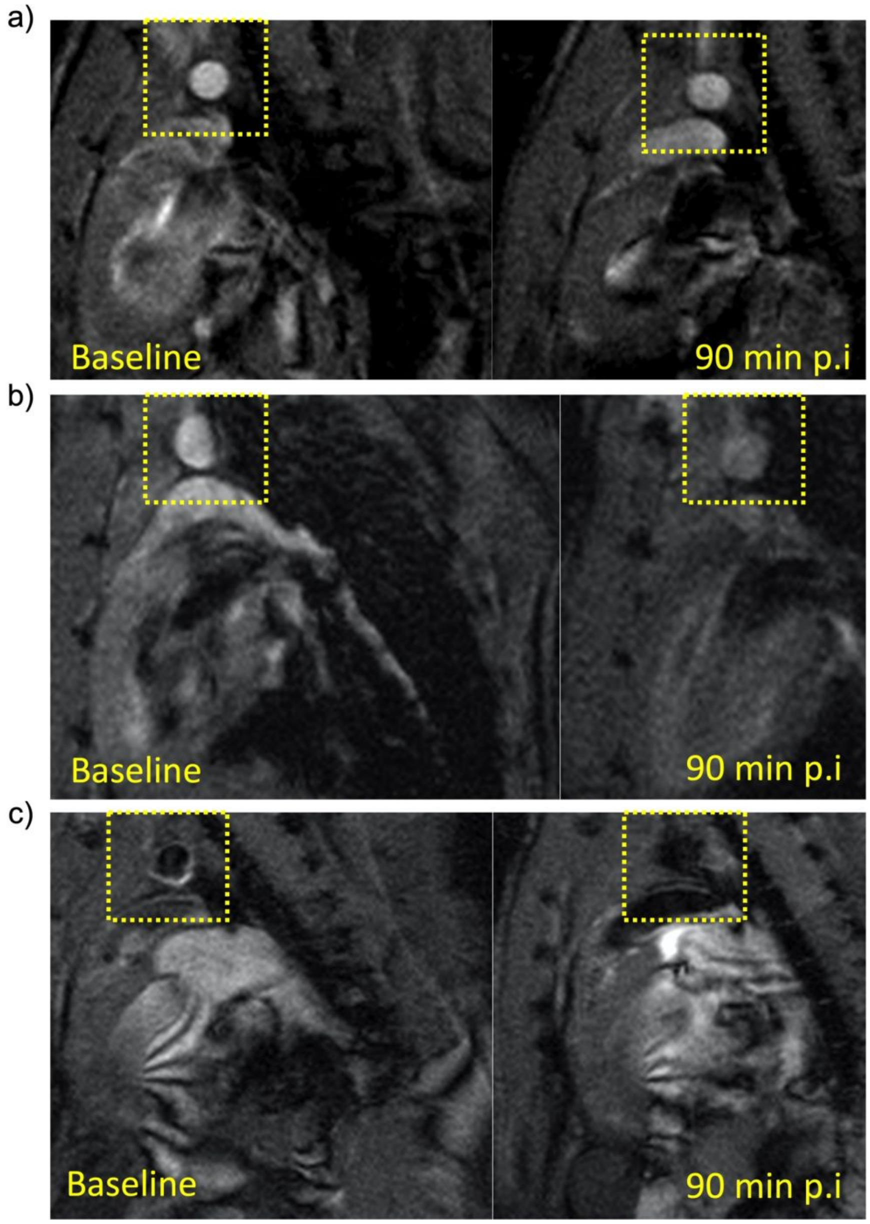

Figure S6. T_1-weighted MRI before (baseline) and 90 min after *i.v.* injection of ^{68}Ga-IONP-citrate for a) Group A ApoE$^{-/-}$ mouse, b) Group B ApoE$^{-/-}$ mouse and c) Group D ApoE$^{-/-}$ mouse.

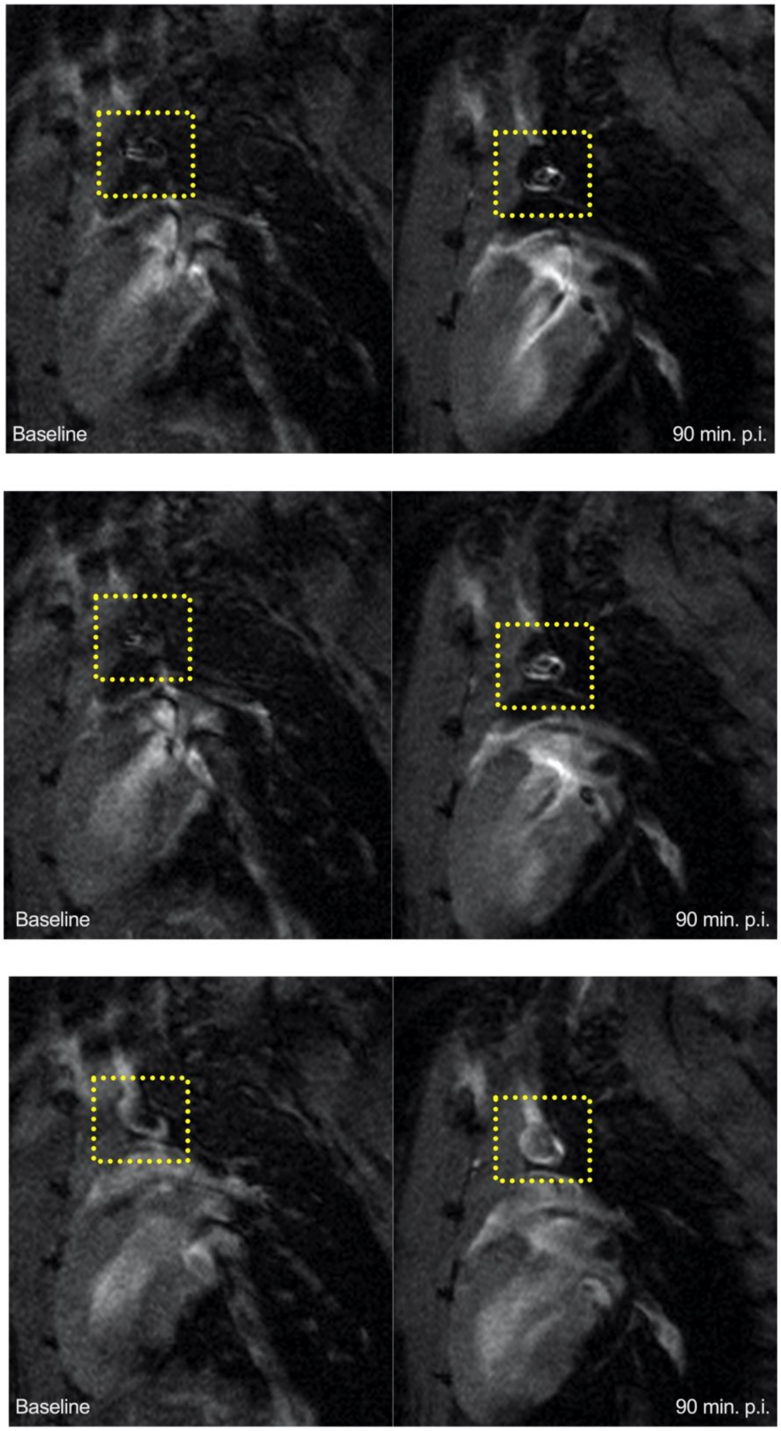
Baseline
90 min. p.i.
Baseline
90 min. p.i.
Baseline
90 min. p.i.

Figure S7. T_1-weighted MRI before (baseline) and 90 min after i.v. injection of HAP-multitag for a Group B ApoE$^{-/}$ mouse.
